# The interrelation between premenstrual syndrome and major depression: Results from a population-based sample

**DOI:** 10.1186/1471-2458-11-795

**Published:** 2011-10-12

**Authors:** Christine Forrester-Knauss, Elisabeth Zemp Stutz, Carine Weiss, Sibil Tschudin

**Affiliations:** 1 Department of Epidemiology and Public Health, Swiss Tropical and Public Health Institute (Swiss TPH), Socinstr. 57, 4051 Basel, Switzerland; 2 University of Basel, Petersplatz 1, 4003 Basel, Switzerland; 3 Department of Obstetrics and Gynaecology, University Hospital Basel, Spitalstrasse 21, 4031 Basel, Switzerland

**Keywords:** Premenstrual syndrome, major depression

## Abstract

**Background:**

Research about the relationship between premenstrual syndrome (PMS) and major depression is limited. This study examined the relationship between moderate to severe PMS and major depression in a population-based sample of women of reproductive age. The objectives of the study were to assess the association between premenstrual syndrome and major depression, to analyse how PMS and major depression differ and to characterise the group of women who report both PMS and major depression.

**Methods:**

Data were obtained from the Swiss Health Survey 2007. Included in the analysis was data from women under the age of 55 without hysterectomy and who answered the questions on PMS symptoms. The population-based sample consisted of 3518 women. Weighted prevalence rates were calculated and relative risk ratios for PMS, major depression and women who reported both PMS and major depression, were calculated with logistic multinominal logit regression.

**Results:**

The prevalence of major depression was 11.3% in women screening positive for moderate PMS and 24.6% in women screening positive for severe PMS. Compared to women without any of these conditions, women who reported moderate to severe alcohol consumption had a lower risk for PMS. Women reporting use of antidepressants, and use of oral contraceptives had a higher risk for major depression compared to women without any of these conditions. Women reporting work dissatisfaction had a higher risk for PMS. A higher relative risk to report both PMS and major depression compared to women without PMS or major depression was related to factors such as high psychological distress, low mastery, psychotropic drug consumption, and low self-rated health.

**Conclusions:**

The results suggested that women who suffer from both PMS and major depression are more impaired compared to women with only one disorder. The results further indicated that PMS and major depression are different disorders that can, however, co-occur.

## Background

Premenstrual syndrome (PMS) and premenstrual dysphoric disorders (PMDD), as a severe form of premenstrual syndrome, have shown to be associated with several psychological conditions, such as reduced psychological wellbeing [1-6], mood disorders, particularly depressive disorders [7-14], and exacerbation of depression [15]. There is limited research on the relationship between major depression and premenstrual symptoms from studies with large sample sizes or from population-based studies. Wittchen et al. [16] have indicated a high comorbidity between PMDD and other mood disorders (22.9%) in a community-based study. Similar comorbidity rates were observed for PMS and major depression by Yonkers et al. [9]. In a U.S. population-based study, it was shown that women with menstrual problems were significantly more likely to report depression [17].

The temporal relationship between PMS and major depression has been investigated in several studies yielding conflicting results. Some studies have shown that women with PMS or PMDD have a higher percentage of past major depression than women without PMS or PMDD [10,11,18], while Hurt et al. have reported contradictory results [19]: Although the risk of late luteal phase dysphoric disorder (LLPDD) (the former term for PMDD in the DSM-III-R) was 14% higher in women with a past psychological disorder, it was not increased in women who reported to suffer from a major depression in the past [19]. Breaux, Hartlage and Gehlert [20] concluded in their review that based on existing research it has not been fully proven whether women with PMDD have a higher likelihood to report past major depression.

There is also some evidence that women with PMDD might be at a higher risk to develop major depression in the future than women without PMDD [20,21].

Besides investigating the relationship between the two disorders, examination of whether and how the two disorders can be differentiated from each other is also of relevance. For PMDD, the criteria of the DSM-IV require the disturbance not to be merely an exacerbation of the symptoms of another disorder, such as major depression. Nevertheless, a clear distinction between PMDD and depression seems not always as clear as wished, and some symptoms, such as depressed mood, feelings of hopelessness, decreased interest in usual activities, concentration difficulties, lack of energy, change in appetite, hypersomnia or insomnia are included in measures for both disorders [22,23]. Some authors described an overlap of symptoms such as irritability, or mood swings between severe premenstrual syndrome and depression [24]. Others, however, emphasised that despite the intriguing similarities between PMDD and depression, they should be regarded as distinct diagnostic entities [25]. Irritability has been described as a more prominent symptom in women with PMS or PMDD than depressed mood [12,26]. Differences in the dysregulation in the stress axes in women with PMDD and in women with current or past depressive disorders also suggest the two disorders to be distinct [27]. Results of studies about risk factors for depression or PMS suggested that the two disorders might have differing causes. In a longitudinal population-based twin study, it was suggested that genetic and environmental risk factors of premenstrual symptoms and major depression are not closely associated [28]. Premenstrual symptoms seem to be only to a small degree or not at all influenced by familial-environmental factors [28].

To our knowledge, little is known about the group of women who report both major depression and premenstrual symptoms. Comorbidity could be related to higher impairment which would make appropriate treatment for this group of women particularly relevant. Soares et al. [29] have shown that women with PMDD and a history of depression were less educated and reported marital disruption less frequently than women with PMDD and no history of depression.

There is a paucity of data from population-based studies on the association between and the distinction of PMS and major depression, and especially knowledge about the group of women reporting to suffer from both is also limited. The first aim of this study was therefore to assess the prevalence of women reporting both major depression and premenstrual symptoms in a large population-based sample and to analyse how women with PMS and depression differ from each other. A second aim was to characterise women who report both major depression and premenstrual symptoms.

## Methods

### Recruitment and Participants

Data presented in this study was assessed for the nationwide Swiss Health Survey 2007 (SHS), which is conducted every five years since 1992 as a cross-sectional study by the Swiss Federal Office of Statistics. The SHS aims to give information about health status, health related behaviors, prevalence and consequences of diseases, and health care utilization. The data of the SHS are available from the Swiss Federal Office of Statistics upon request. For this study, a permission to analyse the data was obtained (Contract Number 30.23-2008). For the SHS 2007, a random sample of adults living in Switzerland aged 15 years or older was drawn. The assessment consisted of two parts, a telephone interview and an additional written questionnaire. The response rate for the telephone interview was 66% (18760). The written questionnaire was sent to all participants of the telephone interview. It was completed by 80% of the participants who had taken part in the telephone interview (14,432). The questions about premenstrual symptoms were included in the written part of the questionnaire.

The sample for this study was restricted to women under the age of 55 with no history of hysterectomy and who answered the questions on PMS symptoms (N = 3522). Mean age was 35.46 years.

### Assessment of premenstrual syndrome

To assess premenstrual symptoms and to differentiate between women with or without moderate to severe PMS, a slightly modified version of the premenstrual symptoms screening tool (PSST) [22] was used. The modified PSST version used in this study consists of 10 items (instead of originally 14 items) each describing a premenstrual symptom. These items had to be rated on a 4-point Likert scale. The PSST is based on the DSM-IV criteria of PMDD. A German translation of the PSST was used for this study. A German version of the PSST by Bentz, Steiner and Meinlschmidt [30] has recently been shown to be a reliable and valid measure for the screening of premenstrual symptoms. Due to restrictions with regard to the number of items that could be included in the Swiss Health Survey, three of the PMS questions were dropped (decreased interest in work activities, home activities and social activities) or merged into one question (insomnia and hypersomnia). The two questions on interference of symptoms with relationships with co-workers and with family were also merged together. To determine severe PMS, the instructions of the premenstrual symptoms screening tool of Steiner et al. [22] were used as followed: For severe PMS, at least one of the main symptoms (anger or irritability, anxiety or tension, tearfulness or mood swings, depressed mood) had to be severe, at least four of the additional symptoms had to be moderate to severe and one of the interferences (work efficiency, relationships with co-workers or family, social life activities, and home responsibilities) had to be severe [22]. For moderate PMS, at least one of the four main symptoms had to be moderate to severe, at least four of the additional symptoms had to be moderate to severe and one of the interferences had to be moderate to severe.

### Assessment of major depression

Major depression was assessed with the World Health Organization's Composite International Diagnostic Interview Short Form (CIDI-SF) [23]. The CIDI-SF is a brief and reliable screening measure that has been validated by Kessler and colleagues [23] and allows generating DSM-IV diagnoses. To calculate a score, 23 of the 35 items of the interview were used. The score was based on reported type and length of symptoms.

### Statistical Analyses

Chi-square tests and multinominal logit regression were conducted using STATA 10 to compare the four groups: women without major depression or PMS, women with major depression only, women with PMS only and women with both major depression and PMS. The statistical analyses were considered to be significant with p < 0.05. For the chi-square tests weighted dated was used.

## Results

### Characteristics of the sample

Table 1 shows sociodemographic characteristics of the total sample and of the four groups (women without PMS and depression, women with PMS, women with major depression, women with both PMS and depression). No significant differences between the four groups were found in age, but were present in marital status and living arrangements, with the highest proportions of being unmarried and separated/divorced, living alone, and being a single parent in women with major depression. Of the overall sample, 7.2% had a major depression, 10.3% moderate PMS and 3.2% severe PMS.

**Table 1:** Socio-demographic characteristics in women without and with PMS, major depression, and with PMS plus depression (weighted prevalence rates)

	No PMS or depression	PMS Only	Major depression only	PMS plus depression	Total	χ^2 ^
	(*n *= 2848)	(*n *= 413)	(*n *= 197)	(*n *= 60)	(*n *= 3518)	(df)
	%	%	%	%	%	
						
**Age (years)**						
14-25	23.7	24.4	23.1	24.1	23.8	
24-35	27.5	21.2	28.2	24.4	26.7	
34-45	31.8	37.4	30.0	23.4	32.2	
44-55	17.0	17.0	18.8	28.1	17.3	
Total	100	100	100	100	100	9.13 (9)
**Marital status**						
Unmarried	41.8	43.3	46.1	42.2	42.2	
Married	50.6	47.5	41.5	50.0	49.7	
Widowed	0.6	1.3	0.7	0.0	0.7	
Separated/Divorced	7.0	7.9	11.8	7.9	7.3	
Total	100	100	100	100	100	18.95* (9)
**Language regions**						
German-speaking	71.3	63.6	71.6	52.5	70.1	
French-speaking	24.4	30.2	24.4	39.8	25.4	
Italian-speaking	4.2	6.2	4.0	7.8	4.5	
Total	100	100	100	100	100	10.56 (6)
**Nationality**						
Swiss	75.9	70.5	75.0	58.7	74.9	
Other	24.1	29.5	25.0	41.3	25.1	
Total	100	100	100	100	100	4.43 (3)
**Profession**						
Higher-middle management	42.5	38.6	39.5	29.6	41.7	
Office worker	31.9	39.4	29.1	23.2	32.4	
Craftsperson	5.5	4.9	6.8	9.7	5.5	
Labourer	20.1	17.2	24.6	37.5	20.3	
Total	100	100	100	100	100	9.39 (9)
**Education**						
Compulsory school	9.3	9.0	8.0	25.8	9.5	
Secondary school	65.3	66	69.7	59.6	65.5	
College/university	25.4	25	22.4	14.6	25	
Total	100	100	100	100	100	5.86 (6)
**Employment**						
Full time	30.1	26.6	29.0	21.6	29.4	
Part time	45.4	41.1	49.2	39.8	45	
No employment	24.5	32.3	21.8	38.5	25.5	
Total	100	100	100	100	100	7.77 (6)
**Living arrangements**						
Living alone	8.8	10.0	17.0	10.1	9.4	
Couple without child	18.9	14.9	16.5	18.7	18.3	
Couple with child	62	63.5	49.4	62.3	61.5	
Single parent	8.5	9.7	16.2	7.4	9	
Other	1.8	1.9	0.8	1.6	1.8	
Total	100	100	100	100	100	21.78* (12)

*p < .05

### Relationship and differences in women with PMS and major depression

Table 2 shows that 11.3% of women with moderate PMS and 24.6% of women with severe PMS suffered additionally from major depression compared to 6.2% of the women who did not report PMS. Current treatment for depression was reported by 3.4% of the women with moderate PMS and 15.1% of the women with severe PMS compared to 2.4% of women without PMS. Daily consumption of antidepressants was highest in women with severe PMS (23.4%) and lowest in women without PMS (5.8%). The highest percentage of women taking tranquilizers or sleeping pills was in women with moderate PMS (16.0%). Of the women with severe PMS, 35% reported to take any type of psychotropic medication.

**Table 2:** Frequencies of major depression, treatment for depression, treatment for psychological problems, consumption of psychotropic medication, and antidepressants in women with and without PMS (weighted prevalence rates)

	No PMS (*n *= 3045)	Moderate PMS (*n *= 363)	Severe PMDD (*n *= 110)	Total (*n *= 3518)	χ^2 ^(df)
					
	%	%	%	%	
					
**Major Depression**					
Major Depression	6.2	11.3	24.6	7.3	
No Major Depression	93.8	88.7	75.4	92.7	
Total	100	100	100	100	11.58** (2)
					
**Treatment for Depression**					
No	92.1	84.5	73.6	90.8	
Yes, before the last 12 months	3.6	7.9	6.2	4.1	
Yes, within the last 12 months	1.9	4.2	5.2	2.2	
In treatment at the moment	2.4	3.4	15.1	2.9	
Total	100	100	100	100	18.02** (6)
**Consumption antidepressants**					
Daily	5.8	9.7	23.4	7.0	
Once to Several times per week	0.5	0.6	8.1	0.9	
Never	93.7	89.7	68.5	92.1	
Total	100	100	100	100	6.77 (4)
**Treatment psychological problems in the last 12 months**					
Yes	5.7	12.5	19.7	6.8	
No	94.3	87.5	80.3	93.2	
Total	100	100	100	100	14.81*** (2)
					
**Consumption of psychotropic medication**					
None	88.8	73.7	65.0	85.9	
Antidepressants only	3.7	5.4	6.9	4.0	
Tranquilizers/sleeping pills only	4.9	16.0	3.5	6.2	
Tranquilizers/sleeping pills and antidepressants	2.6	4.9	24.6	3.8	
Total	100	100	100	100	17.50** (6)

*p < .05; **p < .01; ***p < .001

Differences in health status and health behaviour between the four groups (women without major depression or PMS, women with PMS, women with major depression, women with major depression plus PMS) are shown in Table 3. They were found to be significant for most of the health status variables. The highest percentage of women taking oral contraception was in the group of women who reported suffering from major depression (37.9%). A significant difference between the four groups was also found in alcohol consumption (p = .006) with 9.2% of women with major depression reporting moderate to severe consumption compared to 3.6% of the women without any of the two disorders. Significant differences were also found in psychotropic drug consumption (p = .000), work satisfaction (p = .0009), mastery (p = .000), self-rated health (p = .000), sleeping difficulties (p = .000) and psychological distress (p = .000). Among the four groups, consumption of antidepressants only was highest in women with major depression only (12.4%). In contrary, consumption of sleeping pills/tranquillizers and antidepressants was highest in women with both conditions, PMS and major depression (35.8%). Of the women with both conditions, 74.4% reported low mastery, 25.8% poor self-rated health, 33.5% high psychological distress, 33.6% strongly agreed to sleeping difficulties, and 27.1% reported to be partly satisfied to extremely dissatisfied with work.

**Table 3:** Health status and health behavior in women without and with PMS, major depression, and with PMS plus major depression (weighted prevalence rates)

	No PMS or depression (*n *= 2848)	PMS only (*n *= 413)	Major depression only (*n *= 197)	PMS plus depression (*n *= 60)	Total (*n *= 3518)	χ^2 ^(df)
	%	%	%	%	%	
						
**Body weight**						
Underweight	6.9	6.9	9.7	7.8	7.0	
Normal weight	74.5	72.8	69.2	71.8	74	
Overweight	13.5	13.3	12.3	9	13.3	
Obese	5.1	7.0	8.8	11.4	5.6	
Total	100	100	100	100	100	4.85 (9)
**Pill as oral contraception**						
No	68	78.4	62.1	65.4	68.8	
Yes	32	21.6	37.9	34.6	31.2	
Total	100	100	100	100	100	9.85* (3)
**Alcohol consumption**						
No consumption	33.5	35.1	19	40.5	33	
Mild consumption	62.9	62.4	71.8	53.9	63.2	
Moderate to severe consumption	3.6	2.6	9.2	5.6	3.8	
Total	100	100	100	100	100	17.99** (6)
**Smoking status**						
None smoker	59.1	51.1	47.3	50.4	57.4	
Former smoker	15.3	22	17.9	18.5	16.3	
Current smoker	25.6	26.9	34.8	31.1	26.3	
Total	100	100	100	100	100	12.53 (6)
**Cannabis consumption**						
Yes	2.5	2.7	6.2	7.4	2.8	
No	97.5	97.3	93.8	92.6	97.2	
Total	100	100	100	100	100	4.11 (3)
**Health orientation**						
Not health oriented	9.7	9.6	17	9.6	10.1	
Health oriented	90.3	90.4	83	90.4	89.9	
Total	100	100	100	100	100	4.06 (3)
**Psychotropic drug consumption**						
None	90.9	78.2	67.3	42.6	86	
Antidepressants only	2.9	4.9	12.4	9.7	4	
Tranquillizer/sleeping pills only	4.6	13.1	7.5	11.9	6.2	
Tranquillizer/sleeping pills and antidepressants	1.5	3.8	12.7	35.8	3.7	
Total	100	100	100	100	100	38.99*** (9)
**Work satisfaction**						
Extremely satisfied	23.5	13.6	16.1	12.1	21.8	
Very satisfied	45.2	41.1	33	39.4	43.9	
Fairly satisfied	21.1	24.4	35.2	21.4	22.3	
Partly satisfied to extremely dissatisfied	10.1	20.9	15.8	27.1	12	
Total	100	100	100	100	100	28.35*** (9)
**Mastery**						
Low	17.4	37.6	41	74.4	22	
Medium	46.9	47.7	38.3	20.3	46.1	
High	35.6	14.7	20.7	5.3	31.9	
Total	100	100	100	100	100	104.00*** (6)
**Physical activity**						
Inactive	11.3	16.6	12.5	8.1	11.9	
Partially active	47.2	45.2	51	56.7	47.4	
Active	41.5	38.2	36.5	35.2	40.7	
Total	100	100	100	100	100	6.35 (6)
**Self-rated health**						
Poor health	0.6	3.8	5.5	25.8	1.7	
Fair health	4.8	8.6	13.6	22.8	6.1	
Good health	94.6	87.6	80.9	51.4	92.2	
Total	100	100	100	100	100	40.23*** (6)
**Sleeping difficulties**						
Not at all	66.8	53.6	49.8	31.4	63.7	
A bit	27.2	29	30.2	35.0	27.8	
Strongly	6.0	17.4	20.0	33.6	8.6	
Total	100	100	100	100	100	46.42*** (6)
**Psychological distress**						
Low	84.2	66.0	55.2	34.3	79.6	
Medium	12.9	23.8	30.1	32.2	15.4	
High	2.9	10.3	14.7	33.5	4.9	
Total	100	100	100	100	100	77.31*** (6)

*p < .05; **p < .01; ***p < .001

### Predictors for PMS and/or major depression

Table 4 shows the results of the multinominal logit regression. Relative risk ratios (RRR) were calculated to compare the four groups (women without major depression or PMS, women with PMS, women with major depression, and women with both conditions) with women without major depression or PMS as reference group. Language region (Italian-speaking) and education were the only socio-demographic factors significantly associated with one of the four groups: women from the Italian-speaking part had a higher risk of reporting PMS than the reference group (RRR 1.84, p < .01). Women with a secondary school (RRR 3.83, p < .05) or a university/college degree (RRR 3.72, p < .05) also had a higher likelihood of reporting PMS than women of the reference group.

**Table 4:** Relative risk ratios (95% confidence intervals) from multinominal logistic regression for women without depression or PMS, women with PMS only, women with major depression only and women with PMS and major depression (n = 2349)

	No major depression PMS		PMS		Major depression		PMS and major depression
		**RRR**	**(95% CI)**	**RRR**	**(95% CI)**	**RRR**	**(95% CI)**
							

**Age**							
14-24	1	1		1		1	
25-34	1	1.14	(0.67-1.94)	1.30	(0.62-2.69)	1.22	(0.30-4.95)
35-44	1	1.35	(0.79-2.3)	1.06	(0.49-2.29)	0.52	(0.11-2.50)
45-54	1	1.04	(0.56-1.93)	0.96	(0.41-2.27)	0.92	(0.18-4.71)
**Language regions**							
German-speaking	1	1		1		1	
French-speaking	1	1.15	(0.86-1.55)	0.74	(0.49-1.12)	0.90	(0.40-2.03)
Italian-Speaking	1	1.84	(1.18-2.89)**	0.43	(0.17-1.07)	0.76	(0.17-3.33)
**Marital status**							
Unmarried	1	1		1		1	
Married	1	0.72	(0.46-1.11)	1.38	(0.73-2.61)	1.08	(0.33-3.50)
Widowed	1	0.31	(0.04-2.40)	1.07	(0.13-9.02)	+	+
Separated/Divorced	1	0.90	(0.54-1.50)	1.26	(0.65-2.46)	2.00	(0.47-8.54)
**Nationality**							
Swiss	1	1		1		1	
Other	1	1.08	(0.73-1.62)	1.44	(0.84-2.48)	1.77	(0.68-4.61)
**Education**							
Compulsory school	1	1		1		1	
Secondary school	1	3.83	(1.36-10.83)*	1.72	(0.62-4.74)	0.73	(0.17-3.06)
College/university	1	3.72	(1.30-10.68)*	1.51	(0.53-4.30)	0.68	(0.15-3.20)
**Living arrangements**							
Living alone	1	1		1		1	
Couple without child	1	0.90	(0.57-1.44)	0.56	(0.30-1.06)	3.07	(0.86-10.97)
Couple with child	1	1.29	(0.81-2.04)	0.58	(0.30-1.12)	1.92	(0.49-7.51)
Single parent	1	0.96	(0.56-1.65)	1.20	(0.62-2.30)	0.85	(0.15-4.70)
Other	1	1.52	(0.53-4.33)	+	+	3.77	(0.31-45.4)
**Pill as oral contraception**							
No	1	1		1		1	
Yes	1	0.67	(0.44-1.00)	1.67	(1.03-2.72)*	1.10	(0.39-3.15)
**Alcohol consumption**							
No consumption	1	1		1		1	
Mild consumption	1	0.78	(0.58-1.04)	1.32	(0.84-2.06)	0.96	(0.41-2.27)
Moderate to severe consumption	1	0.34	(0.13-0.88)*	1.87	(0.79-4.44)	1.30	(0.22-7.51)
**Psychotropic drug consumption**							
None	1	1		1		1	
Antidepr. only	1	1.10	(0.43-2.79)	3.16	(1.40-7.13)**	5.38	(1.40-20.72)*
Tranquillizer/sleeping pills only	1	1.85	(0.95-3.59)	0.70	(0.24-2.06)	1.86	(0.40-8.62)
Tranquillizer/sleeping pills and antidepressants	1	1.18	(0.34-4.10)	2.91	(0.95-8.90)	5.18	(1.13-23.84)*
**Psychological distress**							
Low	1	1		1		1	
Medium	1	1.23	(0.86-1.77)	1.68	(1.05-2.69)*	2.33	(0.89-6.10)
High	1	1.77	(0.97-3.23)	3.32	(1.67-6.57)**	7.97	(2.64-24.01)***
**Mastery**							
Low	1	1		1		1	
Medium	1	0.87	(0.63-1.19)	0.51	(0.33-0.79)**	0.42	(0.17-0.99)*
High	1	0.39	(0.26-0.60)***	0.34	(0.19-0.61)***	0.15	(0.03-0.73)*
**Self-rated health**							
Poor health	1	1		1		1	
Fair health	1	0.81	(0.16-4.09)	0.16	(0.04-0.64)*	0.18	(0.03-1.05)
Good health	1	0.46	(0.10-2.16)	0.08	(0.02-0.28)***	0.06	(0.01-0.33)**
**Sleeping difficulties**							
Not at all	1	1		1		1	
A bit	1	1.25	(0.93-1.69)	1.25	(0.82-1.89)	1.27	(0.54-2.99)
Strongly	1	1.03	(0.62-1.73)	1.41	(0.75-2.65)	0.68	(0.20-2.29)
**Work satisfaction**	1						
Extremely satisfied	1	1		1		1	
Very satisfied	1	1.19	(0.80-1.76)	0.84	(0.50-1.4)	0.78	(0.25-2.48)
Fairly satisfied	1	1.64	(1.07-2.51)*	1.38	(0.8-2.38)	0.67	(0.18-2.46)
Partly satisfied to extremely dissatisfied	1	2.42	(1.51-3.87)***	1.05	(0.54-2.04)	1.70	(0.51-5.63)

+: Not enough cases in this category to analyze; *p < .05; **p < .01; ***p < .001

Women taking oral contraception were at higher risk of suffering from major depression than women with none of the two conditions (reference group) (RRR 1.67, p < .05). Women who reported taking antidepressants were more likely to suffer from depression (RRR 3.16, p < .01) and from depression and PMS (RRR 5.38, p < .05). The consumption of both tranquillizers/sleeping pills and antidepressants, was more likely in women with depression and PMS (RRR 5.18, p < .05). Women who reported having moderate to severe alcohol consumption were at lower risk of screening positive for PMS than the reference group (RRR 0.34, p < .05). Differing associations in alcohol consumption were found for women with PMS (RRR below 1) and women with major depression (RRR above 1).

Women with medium or high psychological distress showed a higher risk of suffering from major depression (medium distress: RRR 1.68, p < .05; high distress: RRR 3.32, p < .01) and an even higher risk of suffering from PMS plus major depression (high distress: RRR 7.97, p < .001) than women of the reference group. High mastery and good self-rated health were protective factors. Women who reported high mastery had a lower risk of screening positive for PMS (RRR 0.39, p < .001), from major depression (RRR 0.34, p < .001) and from both (RRR 0.15, p < .05) than women of the reference group. Good self-rated health was highly protective against major depression (RRR 0.08, p < .001) and PMS plus major depression (RRR 0.06, p < .001). Dissatisfaction at work was a significant risk for PMS (RRR 2.42, p < .001).

## Discussion

Results of this population-based study showed that there was a considerable percentage of women who reported both moderate PMS and major depression (11% of women with moderate PMS) or severe PMS and major depression (25% of women with severe PMS). Women with PMS and women with major depression differed mainly in alcohol consumption, psychotropic drug consumption, oral contraception and work dissatisfaction. Factors that were related with a higher relative risk to report both disorders were high psychological distress, low mastery, psychotropic drug consumption, and low self-rated health.

### Differences in women with PMS and major depression

Women who reported using oral contraceptives were more likely to suffer from major depression than the reference group (women without major depression or PMS). Although not significant, they were also less likely to screen positive for PMS, which also has been found in other studies [31]. These results reflect the controversial and inconsistent results of studies on the effects of oral contraceptives on mood disorders and PMS [32-36]. In clinical practice, it seems therefore important to be aware of a higher possibility of depression in women who are using oral contraceptives and to consider referring women with depressive symptoms taking oral contraception for further assessment or treatment to psychologists or psychiatrists. Work dissatisfaction was a further factor that showed a differing risk in relation to PMS and major depression. High work dissatisfaction was a specific risk for PMS. Kuczmierczyk et al. [37] also found that women with PMS reported more work pressure compared to women without PMS. The results of this study do not allow concluding if PMS influences work satisfaction or if certain strains at work contribute to experiencing PMS symptoms. In patients reporting work dissatisfaction PMS should be taken into account as an associated factor. A higher consumption of antidepressants in women with major depression was expected. Alcohol was the fourth factor that differentiated between women with PMS and women with major depression. Women with moderate to severe alcohol consumption were less likely to report PMS.

### Prevalence of both disorders

The prevalence of women who reported both disorders was comparable with prevalence rates found by Wittchen et al. [16], with slightly more than 20% of the women reporting both disorders. Women who reported suffering from severe PMS showed the highest percentage of major depression, with a significant association between severe PMS and major depression.

The results suggest that the group of women who suffered from both PMS and major depression, were the most impaired group, with women with high psychological distress and consumption of antidepressants or a combination of psychotropic drugs having the highest risk to report both conditions. The high impairment and high psychological distress suggests that for treatment an interdisciplinary approach including psychologists or psychiatrists might be beneficial. Good self-rated health and high mastery were protective factors against both conditions (PMS and major depression together). Soares et al. [29] found that women with PMDD and a history of depression were less educated and reported marital disruption less frequently than women with PMDD and no history of depression. These results were not confirmed in this study. Neither marital status nor educational training was significantly associated with both conditions being prevalent.

When considering the discussion about gender differences in depression, the findings about the association between PMS and depression are also of significance. It has been suggested that reproductive hormones might contribute to the increased risk in women of suffering from depression compared to men [38]. The relatively high prevalence of women reporting both PMS and major depression, as found in our study, sustains the hypothesis of the contribution of reproductive hormones and thus might be an explanatory factor for the higher prevalence of depression in women compared to men.

### Strengths and limitations

A limitation of our study is the cross-sectional design. The results can therefore only be interpreted as associations. No causal relationships can be deduced and the history of the development of symptoms is unclear. We can therefore not distinguish if premenstrual symptoms became more severe due to a major depression or if depressive symptoms got worse due to premenstrual symptoms. Furthermore, we used self-reporting measures which might result in an underreporting of the proportion of women with major depression. Assuming that women with major depression tend not to take part in surveys and may be underrepresented in our sample, the prevalence of major depression and of women reporting both disorders may be lower in our study than in the population. The associations between the predictors and the groups may as a result be rather conservative. However, we do not expect a reporting bias between the two affected groups given the observed pattern of associations. The gold standard for the assessment of PMS and PMDD is prospective daily rating. The cross-sectional design and the used screening tools did, however, not allow this procedure and the validity of the diagnosis PMS in our study is therefore not entirely clear. For this reason, we did not to use the term PMDD, but the terms moderate and severe PMS. Furthermore, without prospective daily rating it is also not clear whether some of the women reporting PMS suffered from premenstrual exacerbation of major depression and might therefore falsely be included in the group of women with PMS. Strengths of our study were the large population-based sample as well as the use of two standardized screening tools in the Swiss Health Survey allowing us to address their relationship with additional indicators of health status and health behaviour, and to control for confounds.

## Conclusions

The results of this study suggest that for the group of women who suffer from both major depression and PMS, treatment might be particularly important and needed given that they have been found to be more impaired than women with one condition only. The risk for low self-rated health was highest in women with both conditions, which indicates that they are not only impaired on a psychological level but also feel physically unwell. As Breaux et al. [20] have stated in their review, more research needs to be done on the comorbidity between major depression and PMDD. The necessary differentiation between the two disorders to assess comorbidity is challenging and comorbidity might therefore not always be detected and adequately treated in clinical practice. However, results of this study suggest that it would be important to do so, to be able to know more about their co-occurrence and offer adequate treatment.

## Competing interests

The authors declare that they have no competing interests.

## Authors' contributions

CFK undertook the statistical analysis, did the literature searches, and wrote the draft of the manuscript. CW contributed to the writing. EZS and ST contributed to the interpretation of the results, the writing, finalizing of the manuscript and provided additional literature. All authors have read and approved the final manuscript.

## Pre-publication history

The pre-publication history for this paper can be accessed here:

http://www.biomedcentral.com/1471-2458/11/795/prepub
